# Dual-Color Fluorescent Hydrogel Microspheres Combined with Smartphones for Visual Detection of Lactate

**DOI:** 10.3390/bios12100802

**Published:** 2022-09-28

**Authors:** Sisi Yang, Ziwen Tang, Yilong Tian, Xinghu Ji, Fubing Wang, Conghua Xie, Zhike He

**Affiliations:** 1 College of Chemistry and Molecular Sciences, Wuhan University, Wuhan 430072, China; 2 Wuhan Research Center for Infectious Diseases and Cancer, Chinese Academy of Medical Sciences, Wuhan 430072, China; 3 Department of Radiation and Medical Oncology, Hubei Key Laboratory of Tumor Biological Behaviors, and Hubei Cancer Clinical Study Center, Zhongnan Hospital of Wuhan University, Wuhan 430072, China

**Keywords:** lactate detection, semiconductor quantum dots (QDs), fluorescent nanoparticles, hydrogel microspheres, visual detection

## Abstract

Since it is difficult for human eyes to distinguish between two identical colors with only <15% variation in brightness, mono-color fluorescent hydrogel microspheres have some limitations in the detection of lactate. Herein, we prepared novel dual-color fluorescent hydrogel microspheres, which can achieve hue transformation. Microspheres were prepared by introducing a fluorescent nanoparticle as the reference signal while CdTe QDs were used as the response signal. We used smartphones with image processing software to collect and analyze data. In this way, the signal of lactate was converted to RGB (red, green, and blue) values, which can be quantitatively read. Within 10 to 1500 μM, the R/G values of the microspheres had a linear relationship with the logarithm of the lactate concentration. Moreover, color cards for lactate detection were prepared, from which the color change and concentration of lactate could be easily read by the naked eye. It is worth mentioning that this method was successfully applied to screen patients with hyperlactatemia.

## 1. Introduction

Fluorometric analysis has the preponderances of high sensitivity, low cost, and a short response time [1,2,3]. The detection of the target mainly relies on a single fluorescence signal response using a fluorescent probe with a single emission wavelength, that is, the change in the fluorescence intensity caused by “fluorescence enhancement” or “fluorescence quenching” [4]. However, the signal of monochromatic fluorescent probes is easily interfered with by external environmental factors such as solvents, excitation light sources, and instruments, which affects the detection of target analytes [5,6]. To make up for this deficiency, ratiometric fluorescent probes were designed by introducing a fluorescent material with another emission wavelength.

The ratiometric fluorescent method can reduce environmental influences and improve the probe stability and sensitivity by providing intrinsic self-calibration of the signal [7,8,9,10,11]. Meanwhile, ratiometric fluorescence sensing leads to change in the color tonality. Compared with the change in the fluorescence intensity presented by monochromatic fluorescent probes, this change in the color tonality is more easily recognized by the naked eye [12]. In order to enhance the sensitivity, nanomaterials that have self-calibration together with the unique optophysical properties were used in the preparation of ratiometric fluorescence sensors [13,14]. In the past few decades, plenty of ratiometric fluorescence sensing systems based on nanomaterials have been reported [15,16,17]. For example, Ghasemi et al. designed a ratiometric fluorescent probe with blue-emitting carbon dots (BCDs) as the internal standard and yellow-emitting CdTe QDs (YQDs) as the sensing fluorophore, realizing the detection of Hg^2+^ [18]. In the presence of Hg^2+^, the fluorescence of YQDs was selectively quenched while that of BCDs was unaffected, resulting in a continuous color change from green to purple and then to blue. Currently, changes in color are mainly identified by the naked eye, but it is difficult for the naked eye to distinguish between very similar colors, which also easily causes visual fatigue [19]. Therefore, researchers chose to use smartphones to help solve these problems due to their strong image resolution [20,21,22]. It is convenient to collect the fluorescence signal by taking photos, analyzing the color of images, and reading RGB (red, green, and blue) values through an image processing application [23,24]. For example, Machado et al. built a colorimetric capillary chip combing capillary microfluidics immunoassay with smartphones and realized the rapid detection of mycotoxins [25]. Li et al. constructed a ratio fluorescence probe and combined it with smartphones to realize the detection of Cd^2+^ [26]. Obviously, the combination of smartphones and the ratiometric fluorescent method is intelligent and sensitive.

Herein, we report dual-color fluorescent alginate hydrogel microspheres for lactate (LAc) detection. Alg@PDCN@QDs-LOx MSs were synthesized by investing lactate oxidase (LOx), red-emitting CdTe QDs, and green-emitting phenyl-doped g-C_3_N_4_ (PDCN) nanosheets into sodium alginate hydrogel (Alg) and crosslinking under the effect of Ba^2+^. QDs acted as the response signal, and PDCN nanosheets were used as the reference signal. Data was collected and analyzed using smartphones, and a color card for lactate detection was prepared. Furthermore, we used commercial monodispersed fluorescent microspheres (MFMs) instead of PDCNs to prepare other hydrogel microspheres (Alg@MFM@QDs-LOx MSs), which were easier to prepare and more practical. This realized the visual detection of lactate, and showed a promising application prospect in the screening of patients with hyperlactic acidemia.

## 2. Materials and Methods

### 2.1. Materials and Reagents

Lactate (90%), urea (99%), uric acid (99%), bovine serum albumin (BSA), and L-glutamic acid were purchased from Macklin Biochemical Co., Ltd (Shanghai, China). Tris(hydroxymethyl)aminomethane (Tris), N-acetyl-L-cysteine, and L-cysteine were purchased from Sigma-Aldrich. BaCl_2_·2H_2_O, KCl, Sinopharm Chemical Reagent Sodium offered sodium borohydride (NaBH_4_) and isopropano. Shanghai Chemical Reagents Company (Shanghai, China) supplied CdCl_2_·2.5H_2_O, Na_2_TeO_3_, and CaCl_2_·2H_2_O. Monodisperse fluorescent microspheres (3 μm) were obtained from Aladdin Industrial Inc. (Shanghai, China). Tianjin Heowns OPDE Technologies, LLC (Tianjin, China) supplied lactate oxidase (90 units/mg). Sodium alginate was purchased from Alfa Aesar. Zhongnan Hospital of Wuhan University offered human serum samples. All the experimental solutions were prepared with ultrapure water, which was obtained from a Millipore water purification system.

### 2.2. Apparatus

An RF-6000 PC spectrophotometer (Shimadzu, Kyoto, Japan) was used to obtain the fluorescence spectra. Under a UV lamp (λ_ex_ = 365 nm), fluorescence images were collected with a Xiaomi 8 smartphone. The RGB values were acquired by the application “Color Recognizer”. JEM-2010 FEF transmission electron microscopy (Japan) was used to capture the transmission electron microscopy (TEM) images. An HITACHI S-4800 electron microscope (Japan) was used to obtain scanning electron microscopy (SEM) images.

### 2.3. Synthesis of Two Kinds of Microspheres 

Using a one-pot hydrothermal method, we prepared CdTe QDs [27]. Phenyl-doped g-C_3_N_4_ (PDCN) nanosheets were fabricated according to the methods in the literature [28]. Alg@PDCN@QDs-LOx MSs were synthesized in the following procedures [29]. Firstly, a solution of 1% sodium alginate was obtained by dissolving sodium alginate in 20 mM pH 8.2 tris solution and stirring at about 25 °C for 20 min until the sodium alginate completely dissolved. Then, CdTe QDs, PDCN, and Lox were added to the above solution under vigorous stirring. The concentration ratio of CdTe QDs, PDCN, and LOx was 1.69 nM: 0.84 μg/mL: 0.06 U/mL. Then, the resulting solution was slowly added to the 0.07 M BaCl_2_·2H_2_O solution at a volume of 60 μL per drop. It was important to ensure that each drop was able to take the shape of an independent microsphere and existed for over 25 min in the BaCl_2_·2H_2_O solution. Finally, the hydrogel microspheres were washed and stored at 4 °C. Similarly, Alg@MFM@QDs-LOx MSs were synthesized through the above methods. The concentration ratio of MFM, CdTe QDs, and LOx was 31.26 μg/mL: 1.53 nM: 0.06 U/mL.

### 2.4. Lactate Detection

In the experiments of lactate detection, we adjusted the pH of lactate to 8.2 by adding NaOH solution. The microspheres were added to 300 μL of lactate solution and incubated at 25 ° C for 12 min. Under the UV lamp, we used the Xiaomi 8 smartphone to capture fluorescence pictures of the microspheres. Afterwards, the RGB values were obtained by the application “Color Recognizer”, and a color card for lactate detection was prepared.

### 2.5. Lactate Detection in Human Serum

For the determination of lactate in human serum, the first step was to dilute the serum samples 10-fold. Then, Alg@MFM@QDs-LOx MSs were added to the diluted samples and incubated for 12 min at 25 °C. Under the UV lamp, the fluorescence color of Alg@MFM@QDs-LOx MSs in different serum samples was compared with the color card to rapidly screen patients with hyperlactic acidemia. 

## 3. Results and Discussion

### 3.1. Principle for Lactate Detection

**Figure 1:**
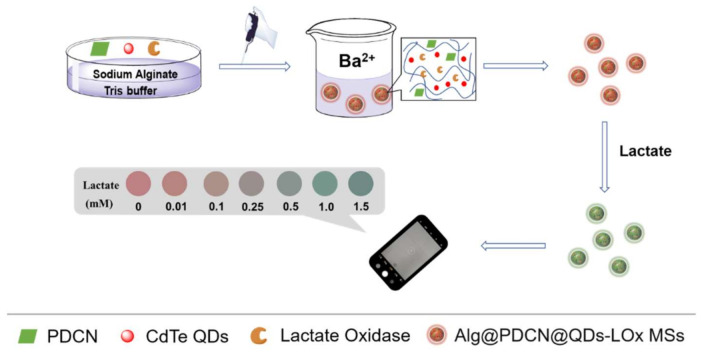
Schematic of the synthesis of Alg@PDCN@QDs-LOx MSs and their application in the detection of lactate.

Firstly, we used the cross-linked method to synthesize Alg@PDCN@QDs-LOx MSs, in which green-emitting PDCN nanosheets were used as reference signals and red-emitting CdTe QDs were used as response signals (Figure 1). In the presence of lactate, lactate oxidase catalyzed lactate to produce H_2_O_2_. The fluorescence of QDs was quenched by H_2_O_2_ while the fluorescence of PDCN was stable, so Alg@PDCN@QDs-LOx MSs showed an obvious fluorescence color change. On this basis, using the mobile phone to take pictures, we established the color card and achieved instant detection.

In order to make the method more practical, commercial monodisperse fluorescent microspheres (MFMs) were purchased to replace the self-made PDCN nanosheets and prepare Alg@MFM@QDs-LOx MSs. When lactate existed, the green-emitting MFMs were undisturbed and the red-emitting CdTe QDs were quenched, presenting a fluorescence color change from red to yellow and then to green. Similarly, a corresponding color card was prepared.

### 3.2. Feasibility Analysis

**Figure 2:**
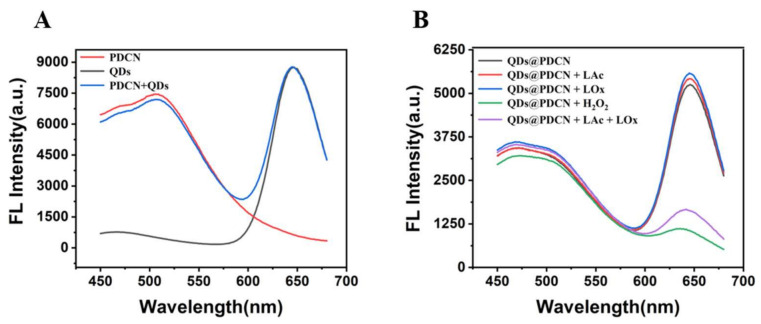
Feasibility test of lactate detection with Alg@PDCN@QDs-LOx MSs. (**A**) Fluorescence spectra of PDCN, QDs, and PDCN + QDs. (**B**) Fluorescence spectra of the CdTe QDs and PDCN mixed solution with tris solution, LOx, LAc, H_2_O_2_, and LAc + LOx. LAc: 1 mM, H_2_O_2_: 1 mM, LOx: 0.06 U/mL.

To prove the scheme, we explored the feasibility of lactate detection with Alg@PDCN@QDs-LOx MSs. In the presence of PDCN, the fluorescence intensity of CdTe QDs remained unchanged (Figure 2A). In the presence of CdTe QDs, the fluorescence intensity of PDCN also remained stable, indicating that PDCN and QDs did not affect each other. Then, the feasibility of using the mixed solution of QDs and PDCN for the detection of lactate was explored, as shown in Figure 2B. When H_2_O_2_ was added or lactate and LOx were added simultaneously, the fluorescence of QDs was effectively quenched. At the same time, the fluorescence of PDCN was nearly invariable, indicating that PDCN as a reference signal and QDs as a response signal could be used for the detection of lactate.

**Figure 3:**
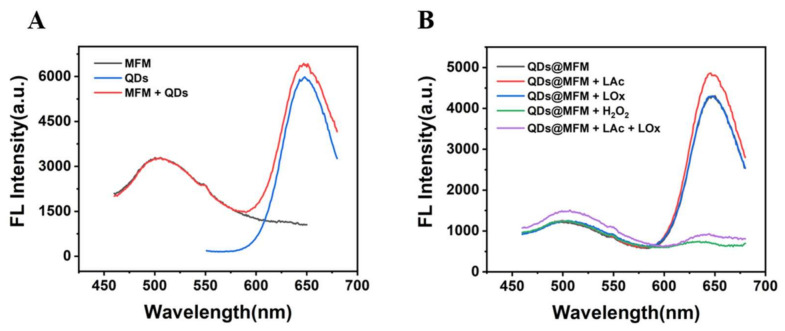
Feasibility test of Alg@MFM@QDs-LOx MSs used for lactate detection. (**A**) Fluorescence spectra of MFMs, QDs and MFMs + QDs. (**B**) Fluorescence spectra of the CdTe QDs and MFMs mixed solution with tris buffer solution, LOx, LAc (pH 8.2), H_2_O_2_, and LAc + LOx (pH 8.2). LAc: 1 mM, LOx: 0.06 U/mL, H_2_O_2_: 1 mM.

Next, we also explored the feasibility of lactate detection with Alg@MFM@QDs-LOx MSs. It was not difficult to find that MFMs and CdTe QDs did not interfere with each other (Figure 3A). When H_2_O_2_ or both LOx and Lac existed, the fluorescence of CdTe QDs could be quenched while the fluorescence of MFMs was stable (Figure 3B), indicating that MFMs as the reference signal and QDs as the response signal could also be used for the detection of lactate.

### 3.3. Characterization of the Fluorescent Microspheres

**Figure 4:**
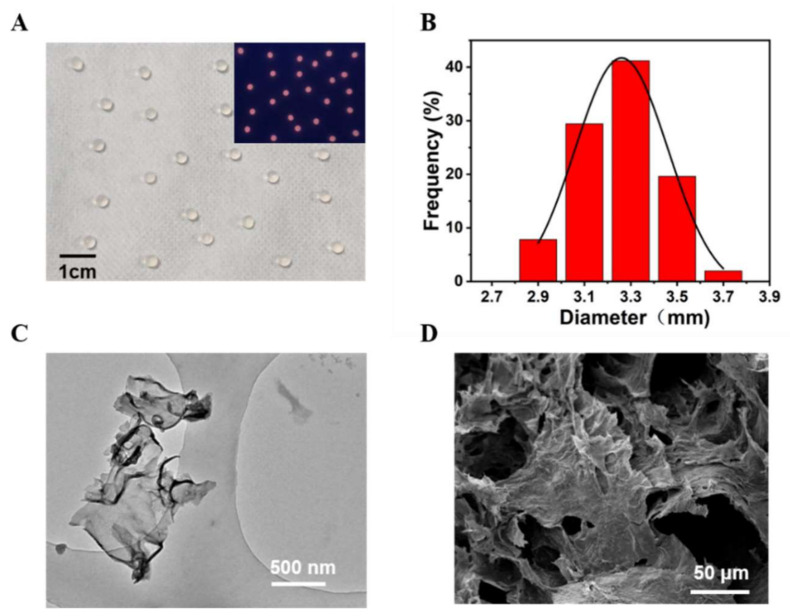
Characterization of Alg@PDCN@QDs-LOx MSs. (**A**) Picture of microspheres under natural light. Inset: Under a UV lamp, a photograph of microspheres. (**B**) The diameter distribution diagram of microspheres. (**C**) TEM image of the PDCN nanosheets. (**D**) SEM image of the microspheres.

Sodium alginate hydrogel was used to embed PDCN, CdTe QDs, and LOx to acquire the Alg@PDCN@QDs-LOx MSs. We found that Alg@PDCN@QDs-LOx MSs showed a uniform spherical shape under natural light and emitted bright red fluorescence under UV light (Figure 4A). The average size of Alg@PDCN@QDs-LOx MSs was about 3.26 ± 0.41 mm (Figure 4B). The PDCN nanosheets were characterized by transmission electron microscopy (TEM), which indicated that the PDCN had a flake structure, as shown in Figure 4C, consistent with the description in the literature [28]. The microspheres were characterized by scanning electron microscopy (SEM), and it was found that Alg@PDCN@QDs-LOx MSs presented a network structure that was multi-layer, as shown in Figure 4D. In addition, CdTe QDs were characterized by TEM (Figure S1 in Supplementary Materials).

**Figure 5:**
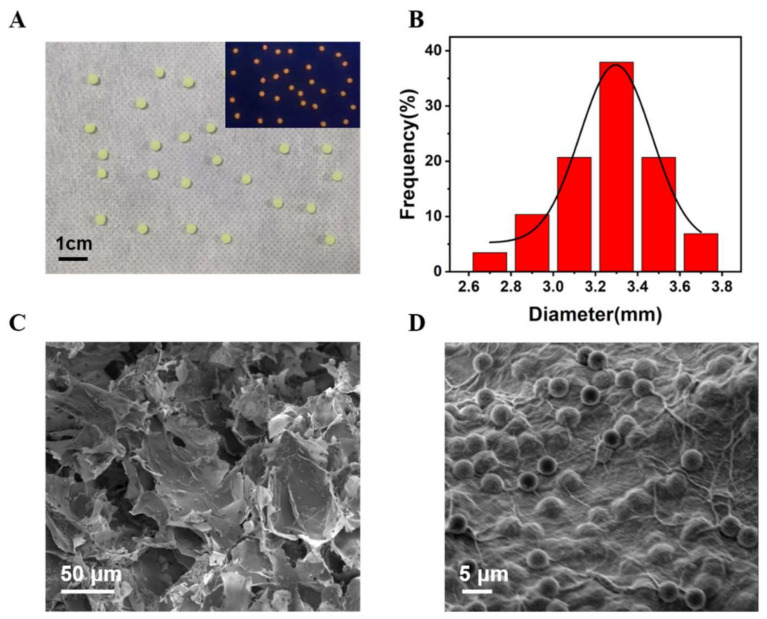
Characterization of Alg@MFM@QDs-LOx MSs. (**A**) Photograph of the microspheres under natural illumination. Inset: Photograph under a UV lamp. (**B**) The size distribution. (**C**) SEM image. (**D**) SEM image of MFMs attached to the Alg@MFM@QDs-LOx MSs.

Under visible light, Alg@MFM@QDs-LOx MSs appeared as structured green spheres and emitted obvious red fluorescence at 365 nm (Figure 5A). The diameter of the microspheres was statistically analyzed (Figure 5B). Meanwhile, its average size was about 3.30 ± 0.23 mm. Alg@MFM@QDs-LOx MSs exhibited a multilayer network structure (Figure 5C), and it was clear that the embedded MFMs were attached to the microspheres (Figure 5D).

### 3.4. Optimization of the Experimental Parameters

For the purpose of enhancing the detection performance, we optimized the experimental conditions, including the ratio of QDs to LOx, ratio of reference materials to QDs, and answering time.

First, we optimized the feeding ratio of QDs and LOx in Alg@PDCN@QDs-LOx MSs. When PDCN was present, the optimized ratio of QDs to LOx was 1.69 nM: 0.06 U/mL (Figure S2). The optimized feeding ratio of PDCN to CdTe QDs was 0.84 μg/mL: 1.69 nM (Figure S3). The best reaction time of Alg@PDCN@ QDS-LOx MSs with lactate was 12 min (Figure S4).

The feeding ratio of QDs and LOx in Alg@MFM@QDs-LOx MSs was also optimized. When MFM was present, the optimized ratio of QDs to LOx was 1.53 nM: 0.06 U/mL (Figure S5). The optimized feeding ratio of MFMs to CdTe QDs was 31.26 μg/mL: 1.53 nM (Figure S6). The reaction reached a plateau at 12 min (Figure S7).

### 3.5. Lactate Detection

We investigated the influence of different lactate concentrations on the fluorescence color of microspheres under the optimized conditions. A color card for lactate detection was prepared by taking photos with smartphones.

**Figure 6:**
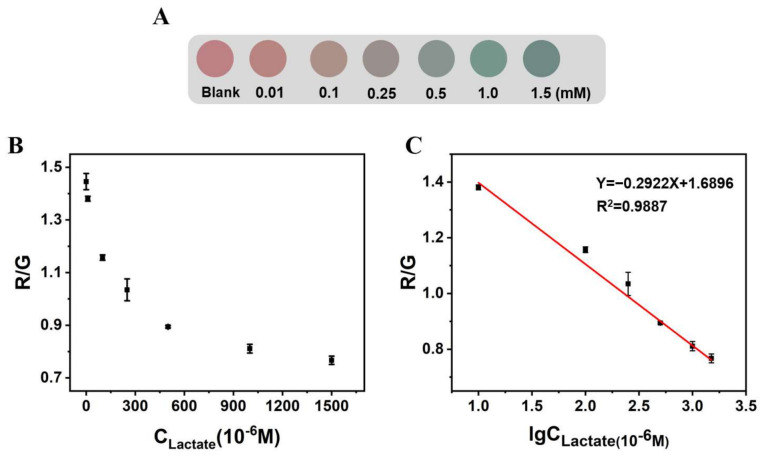
The influence of lactate on the fluorescence color and R/G value of Alg@PDCN@QDs-LOx MSs. (**A**) Influence of lactate on the fluorescence of microspheres. (**B**) The R/G value of Alg@PDCN@QDs-LOx MSs corresponding to different lactate concentrations. (**C**) Linear plot of the R/G value of Alg@PDCN@QDs-LOx MSs with respect to the logarithm of the lactate concentration.

With the addition of the lactate concentration, the color of the microspheres under th UV lamp changed from red to purplish gray then to blue green (Figure 6A). The smartphone image processing application “Color Recognizer” was used to read the RGB values of the microspheres, and the influence of the lactate concentration on the R/G values of the microspheres was explored (Figure 6B). Within 10 to 1500 μM, the R/G values of Alg@PDCN@QDs-LOx MSs had a linear relationship with the logarithm of thee lactate concentration, and the detection limit was 1.39 μM (3σ/s) (Figure 6C).

**Figure 7:**
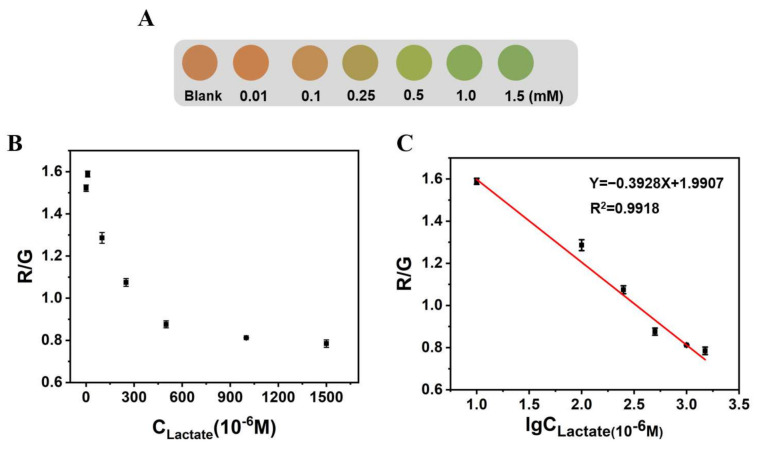
The influence of lactate on the fluorescence color and R/G value of Alg@MFM@QDs-LOx MSs. (**A**) The influence of lactate on the fluorescence of microspheres. (**B**) The R/G value of Alg@MFM@QDs-LOx MSs corresponding to different lactate concentrations. (**C**) Linear plot of the R/G value of Alg@MFM@QDs-LOx MSs with respect to the logarithm of the lactate concentration.

Similarly, we also explored the influence of lactate on the fluorescence color of Alg@MFM@QDs-LOx MSs, and the corresponding color card for lactate detection was prepared (Figure 7). With the addition of the lactate concentration, the fluorescence color of the microspheres changed from orange red and yellow to green. Within 10 to 1500 μM, the R/G values of Alg@MFM@QDs-LOx MSs had a linear relationship with the logarithm of the lactate concentration, and the detection limit was 1.22 μM (3σ/s).

The Alg@MFM@QDs-LOx MSs used for lactate determination exhibited a more obvious fluorescent color in comparison with Alg@PDCN@QDs-LOx MSs because commercial fluorescent microspheres have a longer emission wavelength. When they were excited by UV light, they emitted greener fluorescence, which was more visible to the human eyes. Compared with lactate detection methods reported in other literature, as shown in Table S1, the linear range of this method was wider, and the detection limit was lower.

### 3.6. Selectivity Experiments

The interference of ions, small molecules, and proteins in the biological matrix with Alg@PDCN@QDs-LOx MSs was investigated. After the addition of lactate, the fluorescence color of microspheres turned gray green, and the R/G value decreased significantly (Figure S8). Meanwhile, the fluorescence color and R/G value of the microspheres did not change significantly with the addition of the 10-fold concentration of K^+^, Ca^2+^, Na^+^, glucose, uric acid, cysteine, and glutamic acid and high concentrations of human albumin and γ-globulin (1 μg/mL). Similarly, the Alg@MFM@QDs-LOx MSs showed significant changes only with the increase in lactate (Figure S9). These consequences show that the two kinds of microspheres have sound anti-interference ability and high selectivity to lactate.

### 3.7. Lactate Detection in Human Serum

In order to research the practical applications, we added lactate into human serum samples that had been diluted 10 times to complete the spiked recovery test. When Alg@PDCN@QDs-LOx MSs were used for lactate detection, the recovery percent and RSD values were 99.7–100.2% and 3.4–9.3%, respectively (Table 1). When Alg@MFM@QDs-LOx MSs were used for lactate detection, the recovery percent and RSD values were 90.4–102.5% and 2.0–8.7%, respectively (Table 2).

**Figure 8:**
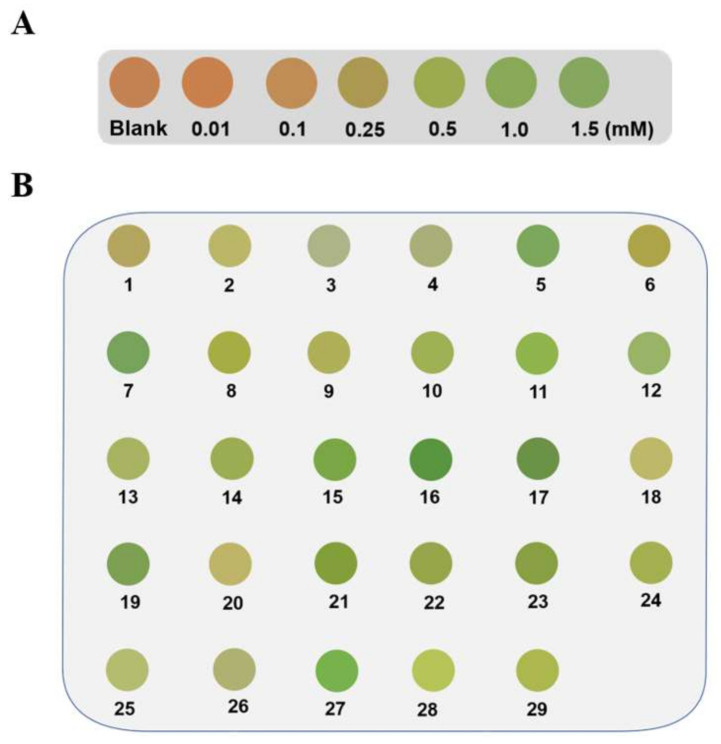
Screening results of Alg@MFM@QDs-LOx MSs for patients who had hyperlactacemia. (**A**) The color card of microspheres for lactate detection. (**B**) Pictures of the microspheres in different serum samples.

Alg@MFM@QDs-LOx MSs showed a better visualization effect in lactate detection in comparison with Alg@PDCN@QDs-LOx MSs. Thus, Alg@MFM@QDs-LOx MSs were opted for to explore the practical application prospect of this method. By placing Alg@MFM@QDs-LOx MSs in a decuple diluted human serum sample, the fluorescent color of the microspheres was observed and compared with the corresponding color card (Figure 8A) to carry out visual screening of patients with hyperlactacemia. Here, 0.25 mM lactate was chosen as the control group as a threshold level. Alg@MFM@QDs-LOx MSs in serum samples from patients with hyperlactacemia emitted green fluorescence, and Alg@MFM@QDs-LOx MSs in serum samples from healthy patients emitted orange-red or yellow fluorescence. As shown in Figure 8B, the microspheres in samples NO. 5, 7, 11, 12, 15, 16, 17, 19, 21, 23, and 27 showed green fluorescence, indicating that the above samples were serum samples of patients with hyperlactacemia, while the microspheres in the other samples showed yellow fluorescence, which were serum samples of normal people. The method was compared with the hospital clinical test results, and the accuracy was 93% (Table S2).

## 4. Conclusions

In summary, we developed two kinds of dual-color fluorescent microspheres (Alg@PDCN@QDs-LOx MSs and Alg@MFM@QDs-LOx MSs) for the visual detection of serum lactate via smartphones. The results revealed that the two kinds of dual-color fluorescent alginate hydrogel microspheres exhibited excellent performances in lactate detection, with a linear range of 101,500 μM. The LODs were 1.39 and 1.22 μM, respectively. In addition, both microspheres showed a good anti-interference ability and lactate selectivity. Comparing the two kinds of microspheres, the preparation process of Alg@MFM@QDs-LOx MSs was simpler. Additionally, Alg@MFM@QDs-LOx MSs showed a more obvious color change and better visual detection effect when used for the detection of lactate. When applied to the screening of patients with hyperlactic acidemia, the accuracy was 93% compared with the clinical results. The lactate detection method established in this work is simple to operate and accurate in the screening of patients with hyperlactic acidemia. This opens the possibility of home testing and timely clinical treatment.

## Figures and Tables

**Table 1:** Recovery of lactate in human serum with Alg@PDCN@QDs-LOx MSs.

Sample	Added (µM)	Found (µM)	Recovery (%)	RSD (%) (n = 3)
Serum	—	121.6 ± 4.1	—	3.4
1	40.00	162.0 ± 15.1	100.2	9.3
2	120.0	237.1 ± 16.6	98.1	7.0
3	200.0	320.6 ± 25.6	99.7	8.6

**Table 2:** Recovery of lactate in human serum with Alg@MFM@QDs-LOx MSs.

Sample	Added (µM)	Found (µM)	Recovery (%)	RSD (%) (n = 3)
Serum	—	143.2 ± 2.8	—	2.0
1	40.00	187.6 ± 13.5	102.5	7.2
2	120.0	237.8 ± 20.6	90.4	8.7
3	200.0	343.6 ± 28.8	100.2	8.4

## Data Availability

All data generated or analysed during this study are included in the published article.

## Supplementary Materials

The following supporting information can be downloaded at: https://www.mdpi.com/article/10.3390/bios12100802/s1, Figure S1: Characterization of CdTe QDs; Figure S2: Optimization of LOx dosage when PDCN was used as a reference; Figure S3: The optimized feeding ratio of PDCN to CdTe QDs_;_ Figure S4: Optimization of the reaction time between Alg@PDCN@QDs-LOx MSs and lactate; Figure S5: Optimization of LOx dosage when MFM was used as a reference; Figure S6: The optimized feeding ratio of MFMs to CdTe QDs; Figure S7: Optimization of the reaction time between Alg@MFM@QDs-LOx MSs and lactate; Figure S8: Selectivity experiments of Alg@PDCN@QDs-LOx MSs; Figure S9: Selectivity experiments of Alg@MFM@QDs-LOx MSs; Table S1: Comparison this developed method with the reported methods for lactate detection [30,31,32,33,34]; Table S2: Comparison of the detection results of lactate from Alg@MFM@QDs-LOx MSs with clinical results from the Zhongnan Hospital of Wuhan University.
