# Supplementary material for: Dual-Color Fluorescent Hydrogel Microspheres Combined with Smartphones for Visual Detection of Lactate
Source: Biosensors (Basel). 2022 Sep 28;12(10):802. doi: 10.3390/bios12100802 (PMC9599631; doi:10.3390/bios12100802)
Supplement: Supplementary file 1 [file biosensors-12-00802-s001.zip › biosensors-1897901-supplementary.pdf]

biosensors

MDPI

Supplementary Material

Dual-Color Fluorescent Hydrogel Microspheres Combined with Smartphones for Visual Detection of Lactate

Sisi Yang [1], Ziwen Tang [1], Yilong Tian [1], Xinghu Ji [1], Fubing Wang [2], Conghua Xie [2,3] and Zhike He [1,2,3,*]

[1] College of Chemistry and Molecular Sciences, Wuhan University, Wuhan 430072, China
[2] Wuhan Research Center for Infectious Diseases and Cancer, Chinese Academy of Medical Sciences, Wuhan 430072, China
[3] Department of Radiation and Medical Oncology, Hubei Key Laboratory of Tumor Biological Behaviors, and Hubei Cancer Clinical Study Center, Zhongnan Hospital of Wuhan University, Wuhan 430072, China
* Correspondence: zhkhe@whu.edu.cn; Tel.: +86-27-68756557; Fax: +86-27-68754067

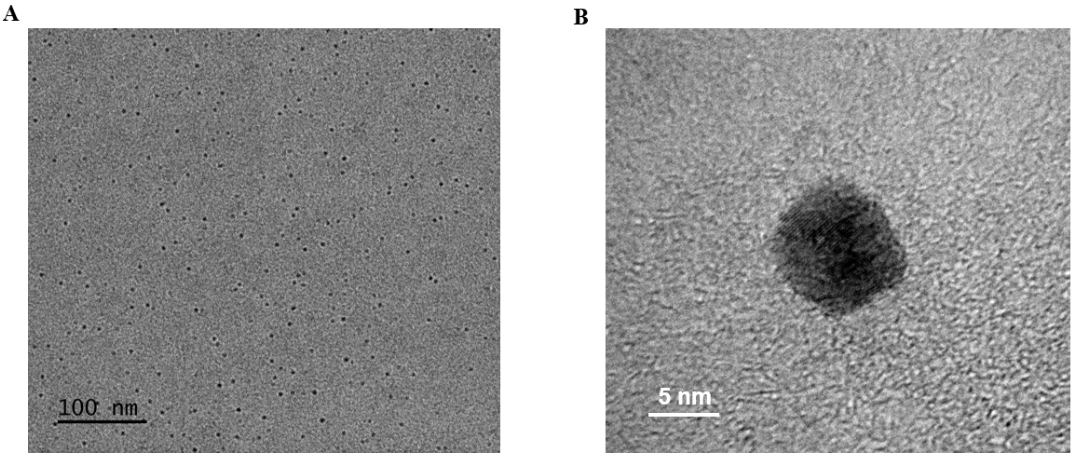

Figure S1. Characterization of CdTe QDs. (A) TEM imaging of CdTe QDs. Scale bar: 100 nm. (B) TEM imaging of CdTe QDs. Scale bar: 5 nm.

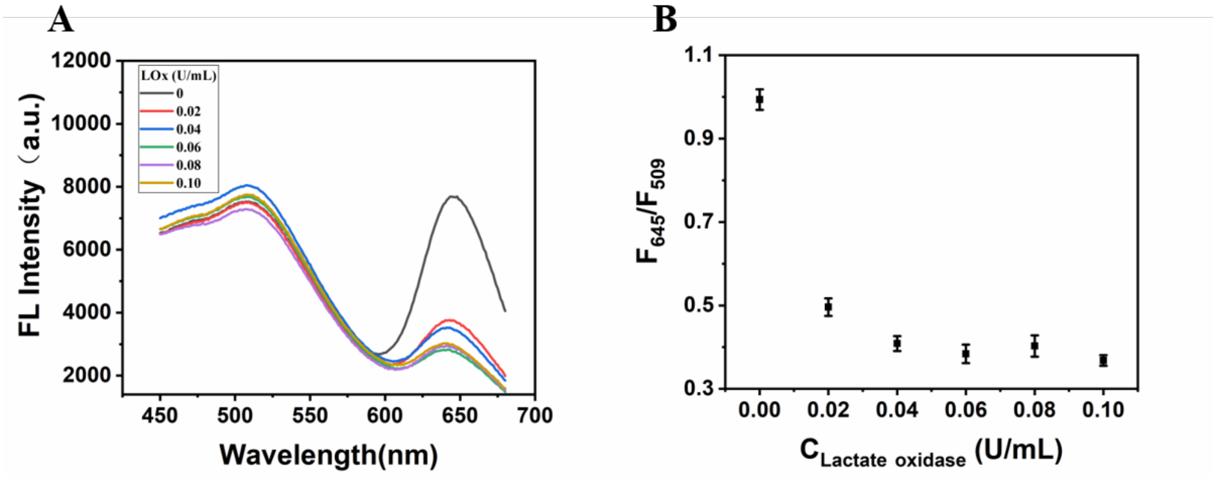

Figure S2. Optimization of LOx dosage when PDCN was used as a reference. (A) Fluorescence emission spectra of the mixed solution of PDCN, CdTe QDs and lactate upon the addition of various concentrations of LOx (0-0.10 U/mL). (B) The effect of concentration of LOx on fluorescence intensity ratio of CdTe QDs to PDCN. Excitation wavelength: 365 nm.

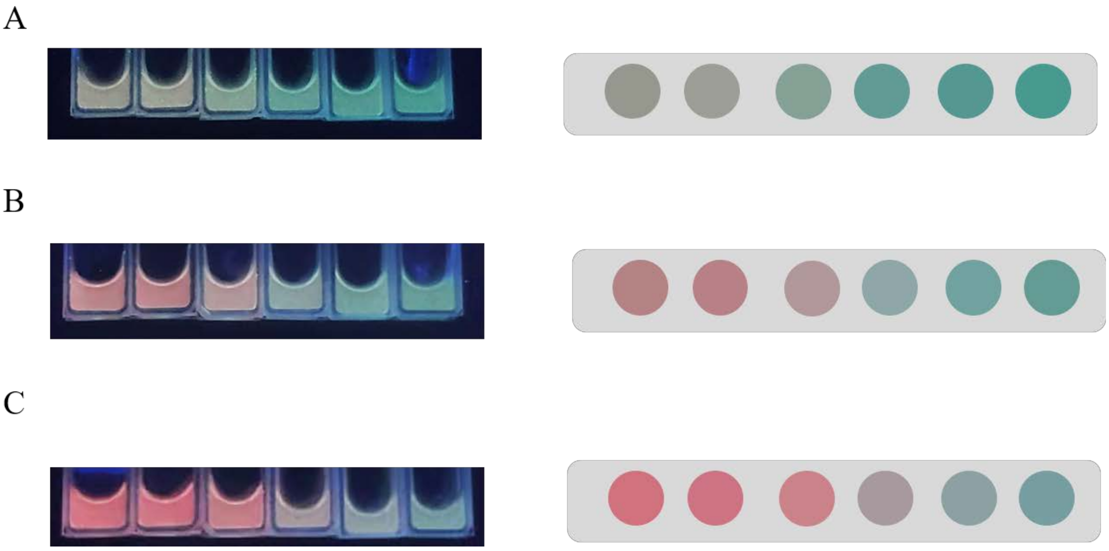

Figure S3. The effect of PDCN/CdTe QDs volume ratio (A: 3:2, B: 3:3, C: 3:4) on fluorescence color of mixed solution when reacting with different concentrations of H_2O_2 (from left to right: 0,0.01, 0.1, 0.5, 1.0, 1.5 mM).

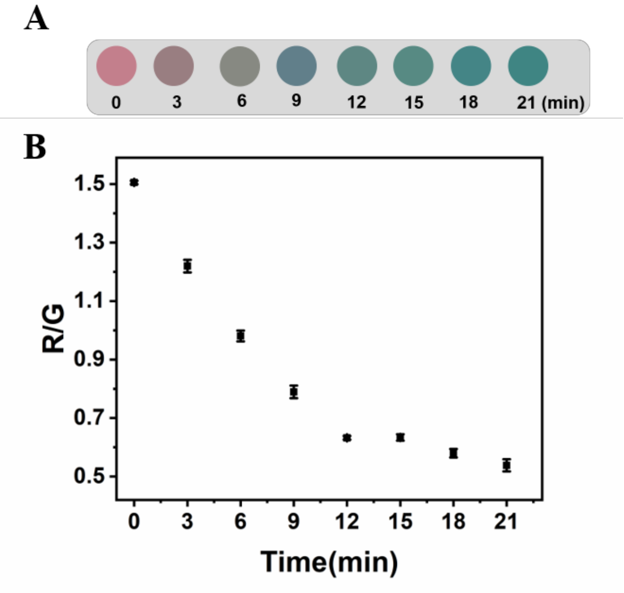

Figure S4. Optimization of the reaction time between Alg@PDCN@QDs-LOx MSs and lactate. (A) The effect of reaction time between Alg@PDCN@QDs-LOx MSs and lactate on the fluorescence color of Alg@PDCN@QDs-LOx MSs. (B) The effect of reaction time between Alg@PDCN@QDs-LOx MSs and lactate on R/G values. Lactate: 1mM, pH: 8.2.

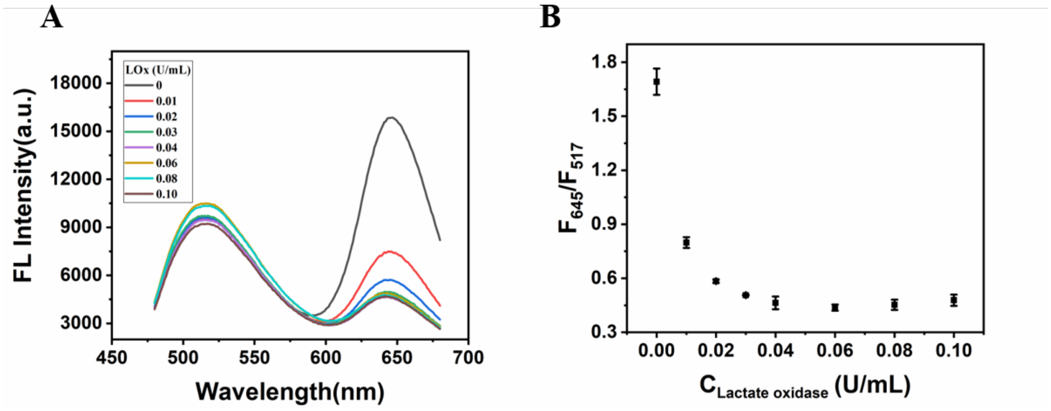

Figure S5. Optimization of LOx dosage when MFM was used as a reference. (A) Fluorescence emission spectra of the mixed solution of MFM, CdTe QDs and lactate upon the addition of various concentrations of LOx (0-0.10 U/mL). (B) The effect of concentration of LOx on fluorescence intensity ratio of CdTe QDs to MFM. Excitation wavelength: 365 nm.

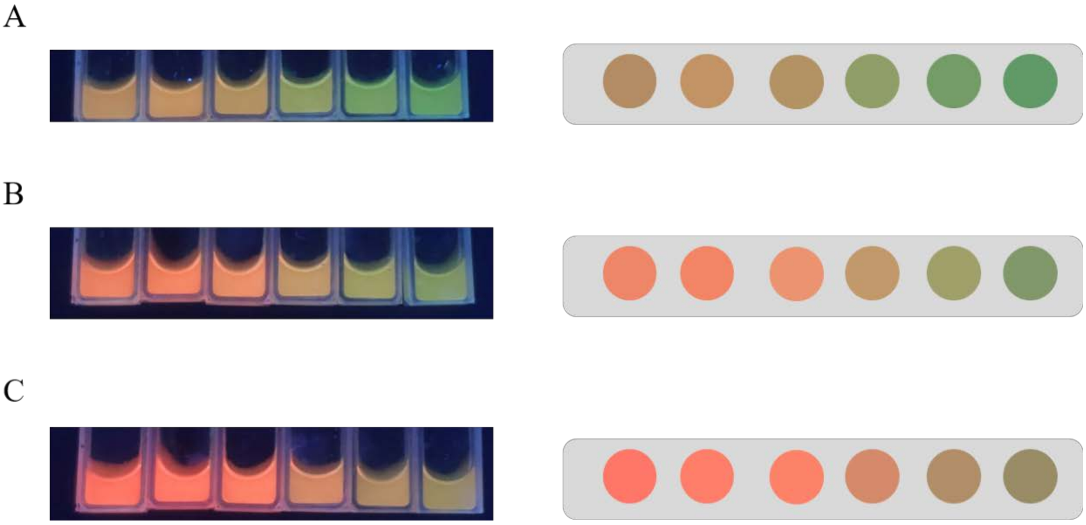

Figure S6. The effect of MFM/CdTe QDs volume ratio (A: 1:1, B: 1:2, C: 1:3) on fluorescence color of mixed solution when reacting with different concentrations of H_2O_2 (from left to right: 0,0.01, 0.1, 0.5, 1.0, 1.5 mM).

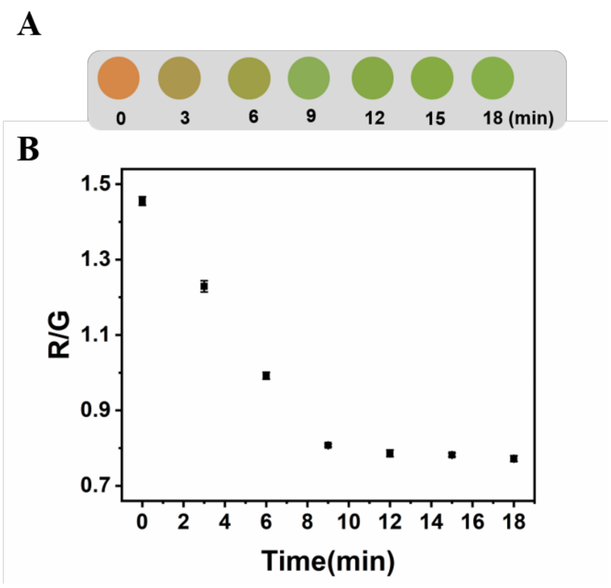

Figure S7. Optimization of the reaction time between Alg@MFM@QDs-LOx MSs and lactate. (A) The effect of reaction time between Alg@MFM@QDs-LOx MSs and lactate on the fluorescence color of Alg@MFM@QDs-LOx MSs. (B) The effect of reaction time between Alg@MFM@QDs-LOx MSs and lactate on R/G values. Lactate: 1mM, pH: 8.2.

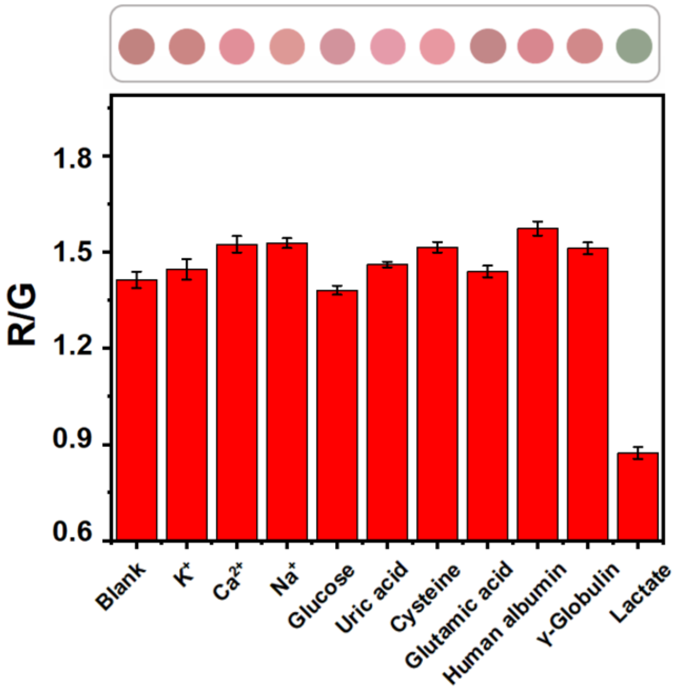

Figure S8. The effect of different ions (10 mM), small molecules (10 mM), protein (1 μg/mL) and lactic acid (1 mM) on R/G value of Alg@PDCN@QDs-LOx MSs.

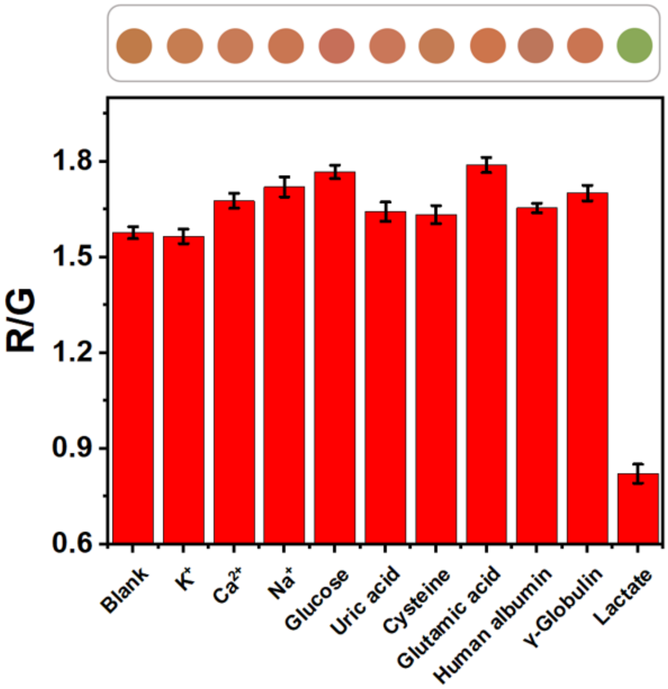

Figure S9. The effect of different ions (10 mM), small molecules (10 mM), protein (1 μg/mL) and lactic acid (1 mM) on R/G value of Alg@MFM@QDs-LOx MSs.

Table S1. Comparison this developed method with the reported methods for lactate detection.

Biosensing materials	Analytical method	Linear range (μM)	LOD (μM)	Reference
—	LC-MS/MS	0.5–100	0.5	[1]
GC/MoS_2/LOx	Electrochemistry	56–770	17	[2]
PB/LOx/chitosan coated gold fiber	Electrochemistry	$0–3×10^4$	137	[3]
PB NPs/g-C_3N_4 NSs	Colorimetry	5–100	2.2	[4]
LOX-PS@PtP*Si@C6 membrane	Ratiometric fluorescence	100–800	60	[5]
Alg@MFM@QDs-LOx MSs	Ratiometric fluorescence	10–1500	1.22	This work

Table S2. Comparison of the detection results of lactate from Alg@MFM@QDs-LOx MSs with clinical results from the Zhongnan Hospital of Wuhan University.

Serum samples	This method	Clinical results	Matching results	Serum samples	This method	Clinical results	Matching results
1	*	*	P	16	+	+	P
2	*	*	P	17	+	+	P
3	+	*	N	18	*	*	P
4	*	*	P	19	+	+	P
5	+	+	P	20	*	*	P
6	*	*	P	21	+	+	P
7	+	+	P	22	+	+	P
8	*	*	P	23	+	+	P
9	*	*	P	24	*	*	P
10	*	*	P	25	*	*	P
11	+	+	P	26	*	*	P
12	+	+	P	27	+	*	N
13	*	*	P	28	*	*	P
14	*	*	P	29	*	*	P
15	+	+	P				

"*" indicates the lactate concentration is in the normal range; "+" indicates the lactate concentration is above the threshold.; "P" indicates the result of this method is consistent with clinical result from the Zhongnan Hospital of Wuhan University; "N" indicates the result of this method is not consistent with clinical result from the Zhongnan Hospital of Wuhan University.

References

1. Jackson, T.C; Zhang, Y.V.; Sime P.J.; Phipps, R.P.; Kottmann, R.M. Development of an accurate and sensitive method for lactate analysis in exhaled breath condensate by LC MS/MS. *J. Chromatogr. B* **2017**, *1061*, 468–473.
2. Parra-Alfambra, A.M.; Casero, E.; Vázquez, L.; Quintana, C.; del Pozo, M.; Petit-Domínguez, M.D. MoS_2 nanosheets for improving analytical performance of lactate biosensors. *Sens. Actuators B:Chem.* **2018**, *274*, 310–317.
3. Wang, R; Zhai, Q.; An, T.; Gong, S.; Cheng, W. Stretchable gold fiber-based wearable textile electrochemical biosensor for lactate monitoring in sweat. *Talanta* **2021**, *222*, 121484.
4. Zhou, D.; Wang, C.; Luo, J.; Yang, M. C_3N_4 nanosheet-supported prussian blue nanoparticles as a peroxidase mimic: Colorimetric enzymatic determination of lactate. *Microchim. Acta* **2019**, *186*, 735.
5. Duong, H.D.; Rhee, J.I. Ratiometric fluorescent biosensors for glucose and lactate using an oxygen-sensing membrane. *Biosensors* **2021**, *11*, 208.
